# Regulation of Hepatitis C Virion Production via Phosphorylation of the NS5A Protein

**DOI:** 10.1371/journal.ppat.1000032

**Published:** 2008-03-21

**Authors:** Timothy L. Tellinghuisen, Katie L. Foss, Jason Treadaway

**Affiliations:** The Scripps Research Institute, Scripps Florida, Jupiter, Florida, United States of America; Mount Sinai School of Medicine, United States of America

## Abstract

Hepatitis C virus (HCV) is a significant pathogen, infecting some 170 million people worldwide. Persistent virus infection often leads to cirrhosis and liver cancer. In the infected cell many RNA directed processes must occur to maintain and spread infection. Viral genomic RNA is constantly replicating, serving as template for translation, and being packaged into new virus particles; processes that cannot occur simultaneously. Little is known about the regulation of these events. The viral NS5A phosphoprotein has been proposed as a regulator of events in the HCV life cycle for years, but the details have remained enigmatic. NS5A is a three-domain protein and the requirement of domains I and II for RNA replication is well documented. NS5A domain III is not required for RNA replication, and the function of this region in the HCV lifecycle is unknown. We have identified a small deletion in domain III that disrupts the production of infectious virus particles without altering the efficiency of HCV RNA replication. This deletion disrupts virus production at an early stage of assembly, as no intracellular virus is generated and no viral RNA and nucleocapsid protein are released from cells. Genetic mapping has indicated a single serine residue within the deletion is responsible for the observed phenotype. This serine residue lies within a casein kinase II consensus motif, and mutations that mimic phosphorylation suggest that phosphorylation at this position regulates the production of infectious virus. We have shown by genetic silencing and chemical inhibition experiments that NS5A requires casein kinase II phosphorylation at this position for virion production. A mutation that mimics phosphorylation at this position is insensitive to these manipulations of casein kinase II activity. These data provide the first evidence for a function of the domain III of NS5A and implicate NS5A as an important regulator of the RNA replication and virion assembly of HCV. The ability to uncouple virus production from RNA replication, as described herein, may be useful in understanding HCV assembly and may be therapeutically important.

## Author Summary

Hepatitis C virus (HCV) is a life-threatening infection afflicting some 170 million people worldwide, and current antiviral therapies are only marginally effective in treating these patients. Clearly, more effective anti-viral drugs for HCV are needed. Of paramount importance to this process is understanding the detailed mechanisms HCV uses to infect cells, replicate the viral genome, assemble progeny virus, and exit the cell. Using genetic mapping, we have identified a single amino acid residue of the HCV NS5A protein that is phosphorylated by host cell kinase, and this modification regulates the production of new infectious virus particles. This modification of NS5A results in the release of some of the viral genome from replicative events, thereby making this material available for progeny virus particle production. We have identified genetic and chemical methods to modulate this event, resulting in our ability to control the production of infectious virus particles in the laboratory. The ability to separate the replication of the virus genetic material and the assembly of new viruses allows us a valuable tool to monitor how this process occurs and, potentially, a novel target for the development of much needed anti-viral drugs.

## Introduction

Hepatitis C virus (HCV) chronically infects nearly 3% of the population of the planet [1]. Persistent virus replication in these individuals often progresses to chronic liver disease, including cirrhosis and hepatocellular carcinoma. Since the discovery of HCV as the causative agent of non-A, non-B hepatitis in 1989 [2], considerable progress has been made in therapeutics, but current anti-virals are still ineffective for the majority of patients. One of the major obstacles to developing new anti-viral strategies is the nebulous nature of many aspects of the HCV lifecycle. One particularly vague area of HCV biology is that of the regulation of the transit of RNAs from active replication to virion biogenesis.

HCV is a member of the *Flaviviridae* family of enveloped, single strand positive sense RNA viruses [3]. The 9.6 kb viral genome contains a single open reading frame encoding a polyprotein that is cleaved co- and post-translationally to yield ten viral proteins [4],[5]. These include the structural proteins (Core, E1 and E2) and the non-structural proteins (p7, NS2, NS3, NS4A, NS4B, NS5A, and NS5B). HCV RNA replication occurs in association with ER-like cellular membranes and requires several viral non-structural (NS) proteins including; NS3, NS4A, NS4B, NS5A, and NS5B, as well as host cell factors [6]. The site of virion assembly is unknown, but recent data has proposed the recruitment of HCV RNA and non-structural proteins by the HCV core protein from the replicase to lipid droplets as an early event in virion assembly [7]. Viral genomes that lack core, or contain mutations in NS5A domain I that block lipid droplet binding, prevent the production of infectious virions [7]. The regulatory events that control these events are not known, but it is clear that productive virus assembly requires the NS5A protein. The complexity of intracellular events associated with HCV infection is staggering, with RNA involved in active RNA replication on positive and negative strand templates, serving as message for translation of viral proteins, and serving as a substrate for progeny virus assembly. These events presumably take place in functionally, and perhaps physically, distinct sub-cellular compartments and require different viral and host factors. There is little information of what regulates these processes to avoid functional conflicts, but the HCV NS5A protein is an attractive candidate for at least some of these roles.

**Figure 1:**
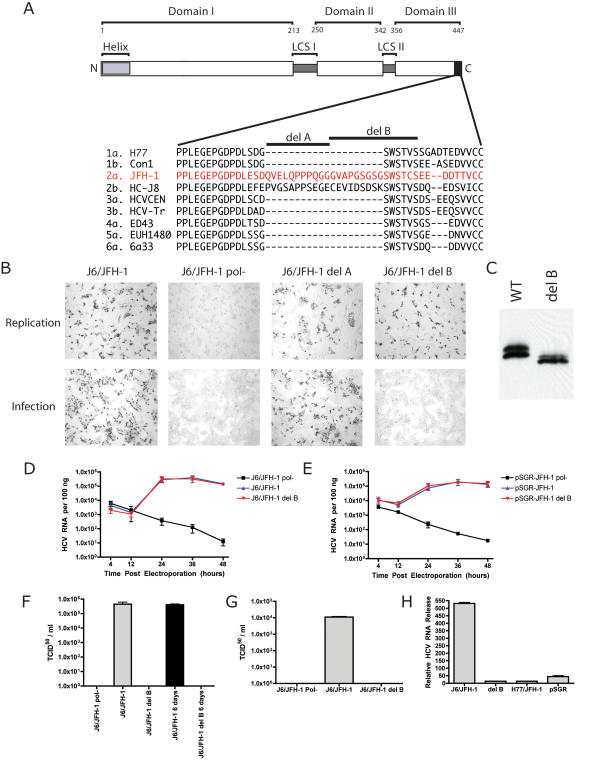
A Deletion in NS5A Domain III Disrupts the Production of Infectious Virus. A). Schematic representation of the domain organization of NS5A indicating the locations of the three domains and low complexity domain connectors (LCS). The location of the membrane anchoring helix is also shown. Numbers indicate amino acid positions on the Con1 genotype 1b NS5A sequence. Sequences of the C terminus of NS5A indicated by the black box on the schematic are shown below for representative sequences of major HCV genotypes. The sequences shown are 1a H77 (AF011753), 1b Con1 (AJ238799), 2a JFH-1 (AB047639), 2b HC-J8 (D10988), 3a HCVCEN (X76918), 4a ED43 (Y11604), 5a EUH1480 (Y13184), and 6a 6a33 (AY859526). B). IHC analysis of cells electroporated with indicated viral RNAs (top of image rows) for NS5A 48 hours post electroporation (top row of panels) or 48 hours post infection (bottom row of panels). C). Western blot of wild type (WT) and deletion B (del B) NS5A from cells electroporated with replication competent full-length genomes shown in B and D. D). RNA replication 48 hour time course of J6/JFH-1 (blue line), J6/JFH-1 deletion B (red line), or J6/JFH-1 pol- (black line) as determined by real time PCR analysis. E). RNA replication 48 hour time course of indicated subgenomic replicon RNAs. pSGR-JFH-1 (red line), pSGR-JFH-1 deletion B (blue line), and pSGR-JFH-1 pol- (black line). F). Infectivity release 48 hours (gray bars) and 6 days post (black bars) electroporation from indicated viral RNA electroporated cells in units of tissue culture infectious dose 50% value per milliliter. G). Infectivity released from cells via freeze-thaw treatment 48 hours post electroporation from indicated viral RNA electroporated cells. H). Relative HCV RNA release from cells electroporated with the indicated HCV constructs. Values reported are arbitrary units normalized to the RNA release by a non-replicating J6/JFH-1 pol-.

NS5A is an absolutely required phosphoprotein component of the replicase, but little is known about its function. NS5A is a zinc metalloprotein organized into three discreet domains (Figure 1A) [8]. The crystal structure of domain I suggests the functional form of NS5A is a dimer, with a large putative RNA binding groove located at the interface of the monomers [9]. The interaction of NS5A with RNA is thought to be critical for the function of this protein in RNA replication [10]. The region of NS5A required for association with lipid droplets, a prerequisite of virion production, is located in domain I [7]. No structural information has yet been obtained for domains II or III. These regions are genetically variable among HCV isolates and can tolerate insertions and deletions without disrupting RNA replication [11]–[15]. Recent genetic mapping has shown many residues in domain II are essential for RNA replication, but domain III is dispensable [15]. Despite having no role in RNA replication, regions of sequence conservation exist in domain III, suggesting a function in the HCV lifecycle. NS5A localizes to the site of RNA synthesis via an N-terminal amphipathic α-helix membrane anchor [16]–[18]. NS5A, unlike the other NS proteins, can be trans-complemented, suggesting it may have functions outside of the viral replicase [19]. The interaction of NS5A with numerous host-signaling pathways has been described, suggesting this protein may modify the host cell environment to a state favorable for the virus, events that may require an extra-replicase form of NS5A [20]. In recent years, the phosphorylation of NS5A has become a major focus area, as this modification may modulate the activity of NS5A in viral and cellular processes.

NS5A is phosphorylated on multiple serine and threonine residues and exists in hypophosphorylated (56 kDa) and hyperphosphorylated (58 kDa) forms [21]. The identification of which of the many potential phosphoacceptor sites in NS5A are relevant to these forms is not known. Nonetheless, evidence has accumulated suggesting that hypophosphorylation primarily targets residues in domains II and III, whereas hyperphosphorylation sites tend to cluster in and around domain I (reviewed in [22]. Cell culture adaptive mutations that increase RNA replication tend to decrease hyperphosphorylation, suggesting NS5A phosphorylation may regulate replication [23]. Compounds selected to decrease the hyperphosphorylation of NS5A increase RNA replication, and these compounds have been shown to function as potent and specific casein kinase 1α (CKIα) inhibitors [24]. CKIα requires pre-phosphorylation of residues near sites of phosphorylation in NS5A for productive modification, suggesting that other kinases also play a role in NS5A phosphorylation [25]. This effect on replication may be due to the differential association of phosphoforms with the host hVAP-A protein, an essential replicase component [26],[27].

A host of other kinases have been shown to target NS5A in heterologous expression/screening systems, including AKT, p70s6K, MEK, MKK1, and CKII [22]. Perhaps the best-characterized NS5A kinase activity, after that of CKIα, is casein kinase II (CKII), with a number of biochemical experiments implicating casein kinase II (CKII) as an NS5A directed kinase [28]–[30]. In vitro phosphorylation of purified NS5A by CKII incorporates 5 moles of phosphate per mole of protein, suggesting at least five sites exist in NS5A [29]. Low-resolution mapping studies have implicated residues in domain III as the predominant sites of CKII phosphorylation (Kim et al., 1999). Further experiments have indicated CKII is likely involved in basal phosphorylation of NS5A, as hyperphosphorylated NS5A is not produced by CKII treatment in vitro [25]. The mutation of a number of potential CKII acceptors does not alter RNA replication [11]. Nonetheless, a body of evidence exists implicating CKII as an NS5A directed kinase. Recent advances in HCV biology have allowed the complete recapitulation of the virus lifecycle in cell culture, allowing the assessment of the function of NS5A phosphorylation in virion production [31]–[34].

In this study we identify a deletion within domain III of NS5A that disrupts the production of infectious HCV virions without altering the replication of HCV RNA. Mapping studies suggest that a single serine residue in a consensus CKII phosphorylation motif is important for the observed phenotype, and phosphomimetic mutants at this position suggest this residue represents a phosphorylation dependent switch controlling virion production. CKII chemical inhibition and siRNA silencing impair infectious virus release for viruses with a serine residue at this position, whereas those with the aspartic acid substitution are insensitive to these treatments. Furthermore, the over expression of CKII increases virus production for serine but not aspartic acid bearing viruses. These results implicate NS5A as a CKII phosphorylation dependent regulator of particle assembly.

## Results

### Deletions in the NS5A protein impair the production of infectious virions

Our previous mapping experiments in the context of the subgenomic replicon system, and the work of others, suggested that domain III of NS5A was not required for RNA replication [11]–[15]. We hypothesized that this region might have a function in virus particle production, at the level of RNA release from the replicase for virion production. This hypothesis is supported, at least in part, by the recent demonstration that the localization of NS5A to lipid droplets is an essential prerequisite for virion production [7]. We generated a panel of deletions within the domain III region of NS5A of the cell culture infectious genotype 2a J6/JFH-1 virus clone to assess the importance of domain III in virion production, including deletion A (amino acids 432–442) and deletion B (amino acids 443–457) (Figure 1A). The preliminary analysis of these two deletions in RNA replication and infectious virus production by an immunohistochemical (IHC) assay is shown in Figure 1B. In this assay in vitro transcripts of various HCV genomic RNAs are generated and electroporated into the highly permissive human hepatoma cell line, Huh-7.5 [35]. Following a 48 hour incubation, the supernatants are harvested, filtered, and used to infect naïve cells. Following an additional 48 hours, cells are fixed and IHC staining for the presence of the NS5A is performed. Wild type virus (Figure 1B, J6/JFH-1), is capable of replication and production of infectious virus, as evident by the presence of NS5A staining in cells in both the electroporated and infected cells. A viral RNA with a lethal lesion in the HCV RNA polymerase (J6/JFH-1 pol-) coding sequence is incapable of replication and infection. Deletion A generated results very similar to the wild type virus, with replication and infection occurring at levels comparable between these two constructs. Deletion B, although showing RNA replication levels similar to that seen for the wild type J6/JFH-1 construct, did not generate any detectable infectious virus production (Figure 1B, last column of images), indicating deletion B could disrupt infectious virus production, while not altering RNA replication.

A more quantitative approach than NS5A IHC was used to better characterize the deletion B virus. For these experiments, HCV RNA transcripts corresponding to wild type J6/JFH-1, J6/JFH-1 pol-, and J6/JFH-1 deletion B were generated and electroporated into Huh-7.5 cells. The cells were plated and following a four hour recovery time, total cellular RNA was harvested in a time course experiment spanning 48 hours. HCV RNA was detected in these samples by quantitative real-time reverse transcription PCR using HCV specific probes and primers. Human GAPDH mRNA was also measured for purposes of data normalization. Figure 1C shows the RNA replication kinetics for J6/JFH-1 and J6/JFH-1 deletion B are identical, indicating deletion B does not affect HCV RNA replication. As lateral spread of virus infection in the J6/JFH-1 electroporated cells, and not in the J6/JFH-1 deletion B cells, could possibly skew results in this type of experiment, we further analyzed the effect of deletion B on RNA replication in the context of the non-infectious HCV 2a replicon, pSGR-JFH-1. Figure 1D shows that RNA replication was not altered for deletion B bearing RNAs in the context of non-infectious HCV replicons. From these data it is clear than deletion B does not alter HCV RNA replication. Turning to the production of infectious virus, we electroporated J6/JFH-1, J6/JFH-1 pol-, and J6/JFH-1 deletion B RNAs into cells, harvested supernatants after 48 hours, and titered the infectious virus in these supernatants by a limiting dilution assay. Viral titers are shown in Figure 1 E in units of 50% tissue culture infectious units per milliliter (TCID_50_/ml). J6/JFH-1 yielded a TCID_50_/ml titer of 5.5×10^5^ and the lethal pol- lesion prevented production of infectious particles. J6/JFH-1 deletion B, despite having RNA replication levels indistinguishable from J6/JFH-1, failed to produce any detectable infectious virus in this assay. To determine if deletion B simply delayed the kinetics of virus production, we incubated electroporated cells for six days post RNA delivery and titered the output of infectious virus from these cultures. We saw no infectious virus production from cells electroporated with deletion B RNA from these six day cultures (Figure 1E), indicating deletion B did not simply induce a kinetic delay in virion production. Furthermore, we have kept deletion B RNA in the context of a neomycin selectable, bicistronic full-length viral genome (FLneo J6/JFH-1, as described in [31] for more than 5 months with no detectable infectious virus output from these cultures (data not shown).

Only a small fraction of the total HCV non-structural proteins present within a cell are actively involved in the process of viral RNA replication [36]. One can easily imagine a mutant that destabilizes NS5A sufficiently so as to eliminate the non-replicative form of the protein, yet retaining enough trace NS5A protein to allow for productive RNA replication. If the non-replicative excess of non-structural proteins are somehow involved in particle assembly, a simple defect in protein stability could lead to a phenotype similar to what we observe with deletion B. To investigate this possibility we performed Western blots to assay the level of NS5A expression in cells electroporated with replication competent wild-type and deletion B full-length viral genomes. Figure 1C shows the results of this analysis. No obvious defect in the levels of NS5A were apparent, but, interestingly, the phosphoform distribution was altered. Wild-type NS5A was present as an even mixture of hyper- and hypophosphorylated forms, whereas deletion B was present in predominantly the hypophosphorylated form, with a small but detectable amount of hyperphosphorylated protein. This observation, combined with the removal of a number of serine residues in the deletion B NS5A protein relative to the wild type sequence suggested that our mutation disrupted one or more phosphorylation sites.

The lack of detectable infectious virus observed for the J6/JFH-1 deletion B construct raised the question as to where in the HCV life cycle the defect in infectivity release might be located. HCV, by analogy to related viruses, most likely buds virions into the ER or an ER-like compartment, and virions are exported from the cell using the host secretory machinery. It has been shown that significant amounts of infectious HCV virions are present inside the infected cell, and can be liberated by simple repetitive freeze-thaw lysis of the cell [37]. To determine if J6/JFH-1 deletion B generates infectious particles that fail to exit the cell, we performed electroporations of this RNA and the parental J6/JFH-1 RNA, incubated the cells for 48 hours, and lysed the cells by freeze thaw after extensive pre-lysis washing to remove virus released by normal exocytosis. Cell lysates were then titered by limiting dilution on naïve Huh-7.5 cells. J6/JFH-1 pol-, as expected, produced no infectious virus in these experiments (Figure 1F). J6/JFH1 liberated approximately 10^4^ infectious units after freeze thaw. Deletion B failed to release any infectious virus in this experiment, suggesting the defect observed for deletion B was not at the step of virus release from the cell, but at an earlier step in virion biogenesis. We next monitored HCV RNA release into the supernatant of infected cells to determine if these cells release RNA and/or HCV particles that are somehow defective in initiation of infection. Figure 1G presents the results of this analysis, with HCV RNA present in cell supernatants quantified by real-time reverse transcriptase PCR using HCV specific primers and probe and presented as relative RNA release to the level of RNA released by a J6/JFH-1 pol- RNA. As expected, wild type J6/JFH-1 infected cells release RNA into the cell culture supernatant, consistent with the release of packaged infectious particles. Deletion B bearing J6/JFH-1 released approximately 525 fold less RNA than wild type J6/JFH-1. RNA release for J6/JFH-1 deletion B was comparable to the non-infectious H77/JFH-1 chimeric virus construct [31] and less than that observed for a subgenomic RNA, pSGR-JFH-1 [38]. These data suggest that the J6/JFH-1 deletion B either does not assemble virions at all or it may release non-infectious particles lacking the HCV genome. To address these possibilities we next looked at HCV core protein, a component of the HCV virion, in the supernatants of J6/JFH-1 and J6/JFH-1 deletion B electroporated cells using the commercially available Ortho Diagnostics HCV core ELISA assay. The use of this assay in HCV cell culture infection experiments as a means to measure virion release is well documented [39],[40]. Table 1 summarizes core protein released at 24 hours post RNA electroporation. J6/JFH-1 pol- RNA electroporated cells released 21.33 femptomoles/L of core protein, establishing the baseline for a non-replicating viral genome. J6/JFH-1 electroporated cells, in contrast, released 2225 femptomoles/L of HCV core, indicating a robust release of core protein. The J6/JFH-1 deletion B electroporated cells released 58 femptomoles/L, considerably less core than the parental J6/JFH-1 electroporated cells. Cell lysates of J6/JFH-1 and J6/JFH-1 deletion B contained comparable amounts of core protein when analyzed by this ELISA assay, indicating no defect in core protein production existed (data not shown). Collectively, these data suggest the defect in J6/JFH-1 deletion B is at an early step of virion production. No viral RNA, capsid protein, or infectious virus are released from cells replicating deletion B RNA, and infectious virions are not merely trapped within the secretory pathway for this mutant.

**Table 1:** Core protein release.

Sample^a^	Core Released (fmoles / L)^a^
J6/JFH-1 pol-	21.33 (+/−2.52)
J6/JFH-1	2225 (+/−28.48)
J6/JFH-1 deletion B	58 (+/−3.61)

a Values reported as average of three independent experiments in the format “average value (+/−standard deviation)” in units of femptomoles of HCV core protein per liter 24 hours post electroporation.

### Identification of Serine 457 of NS5A as a key determinant for virus production

**Figure 2:**
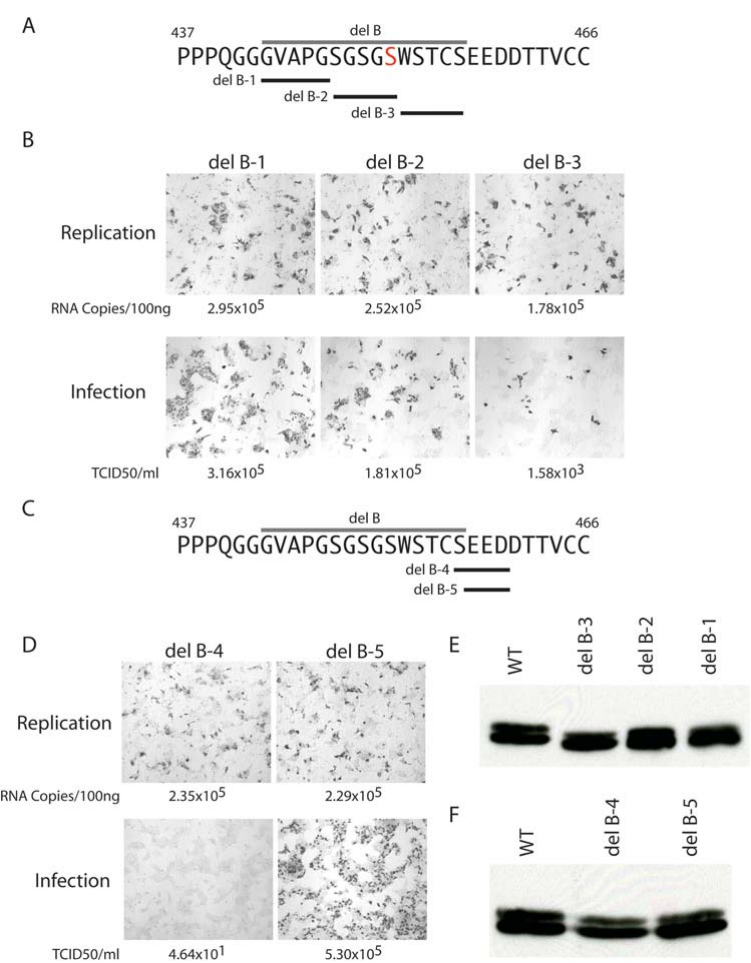
Mapping the disruption of infectious virus production. A). Sequence of JFH-1 NS5A around the region of deletion B (indicated by gray line). Deletions B-1, B-2 and B-3 are indicated by black bars. Numbers refer to amino acid numbering of the mature JFH-1 NS5A protein. Residue serine 452 is shown in red. B). IHC analysis of cells electroporated with indicated deletion bearing viral RNAs (top of image rows) for NS5A 48 hours post electroporation (top row of panels) or 48 hours post infection (bottom row of panels). Numbers beneath each panel are HCV RNA copies per 100 ng of total RNA (top row of images) and infectivity release from titering (bottom row of images). C). Sequence of JFH-1, as described in A, now showing the location of deletion B-4 and B-5 with black bars. D). IHC analysis of deletion B-4 and B-5. Panel layout is as described in B. Numbers below the images are as described in B. E). Western blot analysis of NS5A from cells electroporated with replication competent full-length viral genomes of wild-type (WT) and deletions B-1, B-2, and B-3 (del B1, del B2, and del B3) shown in panel B. F). Western blot analysis of NS5A from cells electroporated with replication competent full-length viral genomes as shown in panel D. Nomenclature is as described in E.

As the preliminary characterization of J6/JFH-1deletion B suggested this region of NS5A was important for the production of infectious HCV virions, we decided to map what residues in deletion B were responsible for this phenotype. We therefore generated three smaller deletions, designated B1 (amino acids 443–447), deletion B-2 (amino acids 448–452) and deletion B-3 (amino acids 453–457), in the context of the wild type J6/JFH-1 virus (Figure 2A). Each of these deletions was capable of robust HCV RNA replication (Figure 2B, top row of images). Deletions B-1, B-2, and B-3 were also capable of producing infectious virus, although deletion B-3 produced considerably less infectious virus than deletion B-1 and B-2 (Figure 2B, bottom row of images). However, none of these deletions was capable of reproducing the complete loss of infectivity release observed with the original deletion B mutant. From these mutants it was clear that none of the residues in deletion B-1 or B2 were essential for virus production. Residues in deletion B-3 clearly have some impact of infectivity release, but not to the extent of deletion B. As deletions B-1, B-2, and B-3 completely span the parental deletion B region, we were puzzled by our inability to map the region of deletion B involved in the disruption of infectivity release. Upon closer examination of the deletions we generated we noticed that generating deletion B-3 positioned an upstream serine residue at position 452 (residue highlighted in red in Figure 2A) in a position analogous to serine 457 that was removed in deletion B-3. This data hinted that having a serine in this position might be important for the production of infectious virus, and also suggested that residues upstream from that serine (deletion B-3 region) might have some impact of infectivity release, as evidenced by deletion B-3's reduced infectivity release. We therefore generated two additional deletions, deletion B-4 (amino acids 457–460, sequence SEED) and deletion B-5 (amino acids 458–460, sequence EED), to evaluate the importance residues near this serine (Figure 2C). Both deletions B-4 and B-5 were capable of efficient replication upon electroporation of these RNAs into cells (Figure 2D). Deletion B-4, removing serine 457 and three downstream residues, failed to generate infectious virus in a manner similar to that observed for the parental deletion B (Figure 1B). Deletion B-4, retaining serine 457 and deleting only the three downstream residues, produced a robust level of infectivity. Collectively, these studies implicate serine 457 as a key residue in the production of infection virus, although the involvement of other residues in this region (specifically in deletion B-3) may affect the efficiency of this process. We have subsequently generated deletions of serine 457 alone, and this also blocks infectivity release (data not shown). We next analyzed the stability and phosphorylation state of our NS5A deletions by Western blotting lysates prepared from cells electroporated with the constructs shown in Figure 2, panel B and D. Figure 2, panels E and F show the results of this analysis. No obvious defects in NS5A stability were evident for any of the mutant NS5A proteins. Interestingly, we observed less hyperphosphorylated NS5A with deletion B-3 and B-4, deletions which reduced infectivity release. This is reminiscent of our observations with the parental deletion B, again suggesting these mutations alter hyperphosphorylation of NS5A.

### Mutations suggest the phosphorylation of Serine 457 regulates RNA replication and infectious virus production

**Figure 3:**
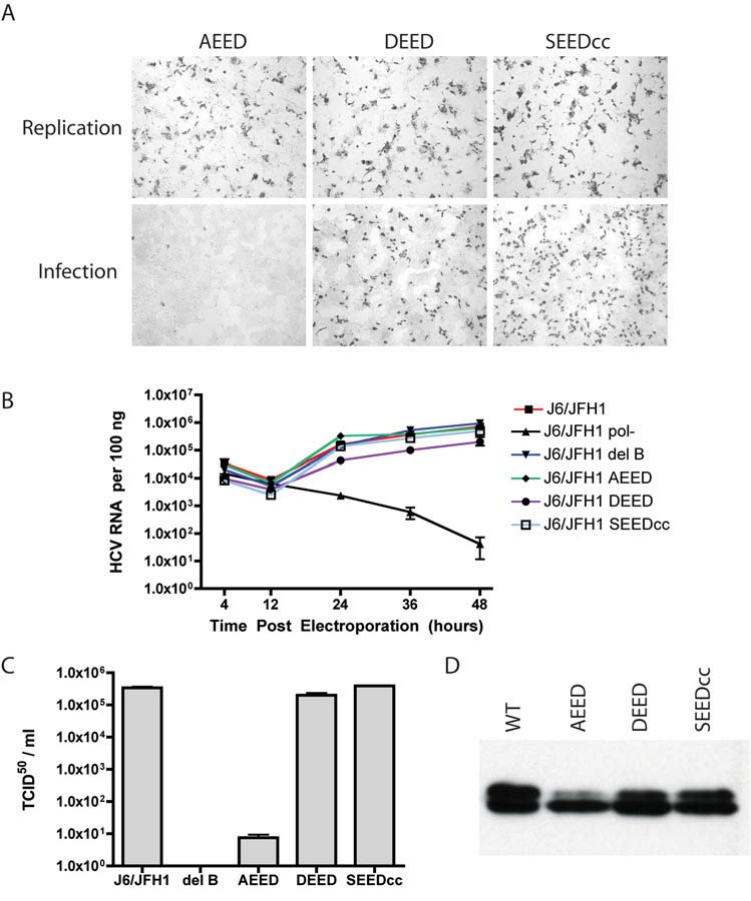
Single amino acid mutations that mimic phosphorylation of serine 457 alter particle assembly. A). IHC analysis of cells electroporated with indicated point mutation bearing viral RNAs. AEED indicates mutation of serine 457 to alanine, DEED is the same residue changed to aspartic acid, SEEDcc is a change of the codon of serine 457 while retaining serine at this position. Rows of images are as described in 1B, 2B and 2D. B). RNA replication 48 hour time course of J6/JFH-1 (red line), J6/JFH-1 deletion B (blue line), J6/JFH-1 pol- (black line), AEED RNA (green line), DEED RNA (purple line), and SEEDcc RNA (light blue line) as determined by real time PCR analysis. C). Infectivity release 48 hours post electroporation from indicated viral RNA electroporated cells in units of tissue culture infectious dose 50% value per milliliter. D). Western blot analysis of NS5A from cells electroporated with replication competent full-length viral genomes as shown in panel A. Nomenclature used is as described in A.

As our mapping studies suggested that serine 457 of NS5A was an important residue for the production of infectious HCV, and the phosphorylation of NS5A by a number of cellular kinases is well documented in the literature, we explored the possibility that serine 457 was a phosphoacceptor site. The web based NetPhosK server strongly predicted serine 457 as a site of CKII phosphorylation, with serine 457 present in an ideal motif for CKII phosphorylation (SEED sequence) [41]. We proceeded with the hypothesis that serine 457 is a phosphoacceptor site, and that this site is a target of CKII. We therefore generated mutations at position 457 in the context of the infectious J6/JFH-1 viral genome, including an alanine substitution (AEED) that is incapable of phosphorylation at position 457, an aspartic acid (DEED) that should mimic phosphorylation at this site, and a serine to serine codon change (SEEDcc) that should be capable of phosphorylation and eliminate any chances that our previous mapping work merely disrupted an RNA packaging signal or other RNA element required for infectious virus production. Figure 3A shows the analysis of these mutants using the IHC NS5A detection assay for replication and infection. The substitution of alanine for the serine at position 457 did not alter RNA replication, but blocked the production of infectious virus and the subsequent infection of naïve cells, although a few infected cells were visible in this experiment. The aspartic acid mutation (DEED) and the serine codon change mutation (SEEDcc) had not obvious effect on RNA replication or infectivity release in this assay. Using the more quantitative real-time PCR based assay, we generated transient RNA replication curves for all of our mutations at position 457 and the wild type and deletion B bearing J6/JFH-1 RNAs (Figure 3B). J6/JFH-1, J6/JFH-1 deletion B, J6/JFH-1 AEED, and J6/JFH-1 SEEDcc RNAs had no measurable defect in RNA accumulation levels or the kinetics of RNA accumulation. The J6/JFH-1 DEED mutant showed a small but reproducible ½ log reduction in RNA levels in this assay. We next looked at the release of infectious virus for these mutant RNAs by titering released virus in the supernatants by limiting dilution (Figure 3C). J6/JFH-1 released approximately 5.5×10^5^ TCID_50_/ml, with the lethal polymerase lesion (J6/JFH-1 pol-) releasing no infectious virions. J6/JFH-1 deletion B, as shown in previous experiments, failed to release any detectable infectivity. The J6/JFH-1 AEED mutant virus released infectious virus at very low levels (about 60–80 TCID_50_/ml), in good agreement with the observation of a few NS5A positive cells in the lower infection panel in Figure 3A, indicating that substitution of serine 457 to alanine inhibited infectivity release by about five orders of magnitude. The low level of infectivity release suggested these infected cells might be harboring revertants. We expanded these serine 457 to alanine infected cultures, extracted RNA, and performed RT-PCR based sequencing on the bulk population. This analysis revealed that the input alanine mutation was retained in the infectious virus produced by this mutant, indicating the requirement of serine 457 may not be absolute, or that other changes elsewhere in the HCV genome may compensate for this mutation (data not shown). We sequenced the entire HCV coding sequence and were unable to identify any additional sequence changes, furthering the idea that a low level of particle production may occur even in the absence of serine 457. The J6/JFH-1 DEED released virus at about 3×10^5^ TCID_50_/ml, indicating aspartic acid was an acceptable substitution at position 457. J6/JFH-1 SEEDcc produced a virus titer similar to that of wild type J6/JFH-1, indicating changes in the HCV RNA sequence at this position were not responsible for the observed reduction in infectious virus production. Analysis of the stability and phosphorylation state of these mutants (Figure 3D) revealed no obvious defect in stability and a loss of some hyperphosphorylated NS5A in the AEED mutant. The reduction of NS5A hyperphosphorylation seems to be common to all of our mutants that are of reduced infectivity release. At first glance it would seem likely that serine 457 is a phosphoacceptor site, and the alteration or deletion of this residue leads to reduced levels of hyperphosphorylated NS5A. However, the situation is more complex, as the DEED mutant also eliminates this serine residue, yet has levels of hyperphosphorylation comparable to wild type NS5A or the many virus release competent mutations. The data suggests that serine 457 phosphorylation is a prerequisite to modification of other phosphoacceptors involved in generating hyperphosphorylated NS5A.

### The casein kinase II inhibitor DMAT disrupts virus production

**Figure 4:**
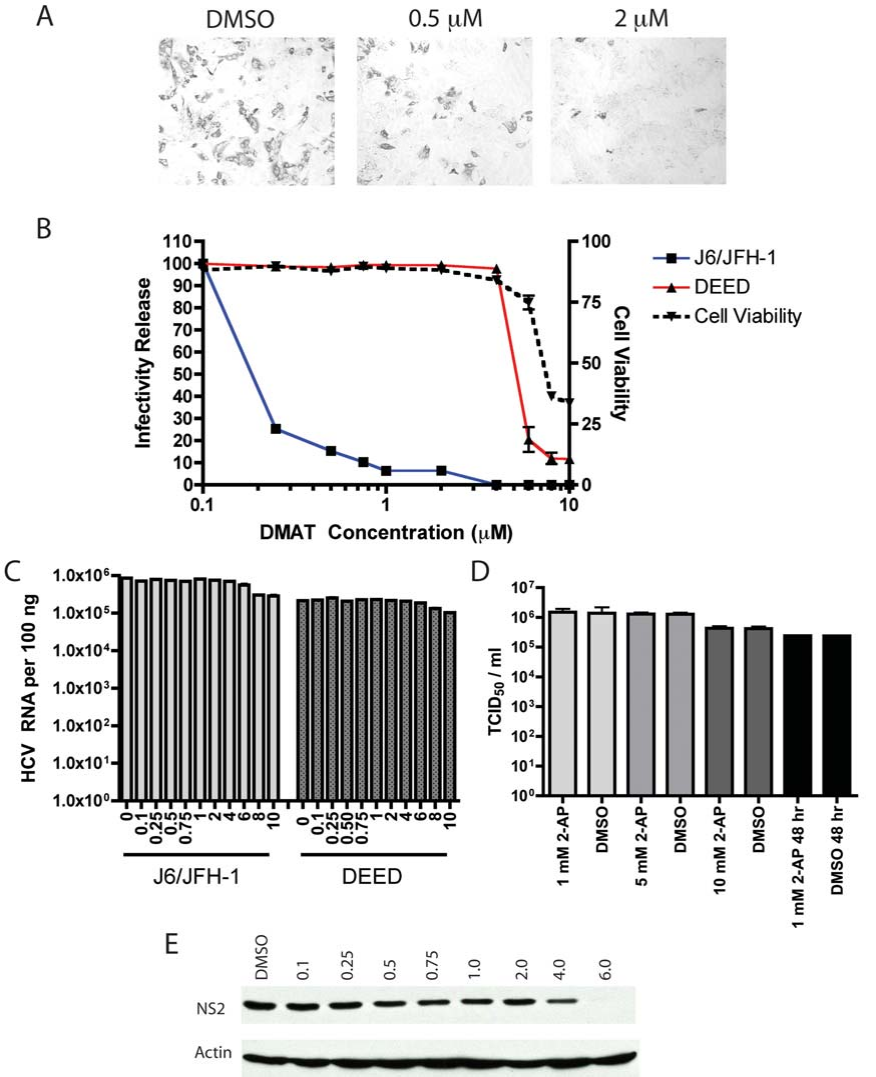
The casein kinase II inhibitor DMAT disrupts the production of infectious virus. A). IHC infectivity assay showing cells 48 hours post infection stained for NS5A expression. Individual images correspond to cells treated with DMSO, 0.5 µM DMAT in DMSO, and 2 µM DMAT 4 hours after electroporation. B). Infectivity release of J6/JFH-1 and J6/JFH-1 DEED 48 hours post electroporation versus dose of DMAT added to cells. Values shown are percent infectivity release of DMSO treated cells receiving identical viral RNAs. Cell viability is percent viable cells of a DMSO only treated control cells as measured by an ATP based cell viability assay. C). HCV RNA replication 48 hours post indicated concentration of DMAT treatment for J6/JFH-1 (light gray bars) and J6/JFH-1 DEED (dark gray bars) RNA electroporations. Bars designated zero are DMSO only treated. D). Treatment of cells with an unrelated kinase inhibitor, 2-aminopurine (2-AP), does not affect infectious virus release. Cells were treated with 2-AP at the indicated concentration or a DMSO control for 1 hour prior to infection. Virus output was then titered 48 hours post infection. In another experiment, 2-AP, was left in the culture medium at 1 mM for the 48 hour course of the experiment (black bars). E). Effect of CKII inhibition with DMAT on NS2 stability. Indicated doses of DMAT were added to cells electroporated with JC1 HCV RNA for a period of 48 hours. Following this treatment, cells were lysed and Western blots for NS2 (top row) and β-actin (bottom row) were performed to look for differences in NS2 stability.

To determine if CKII phosphorylation of serine 457 was important for the process of virion biogenesis in vivo we investigated the use of the specific CKII inhibitor 2-dimethylamino-4,5,6,7-tetrabromo-1H-benzimidazole (DMAT) on infectious virus release. DMAT is a cell-permeable ATP-competitive inhibitor that displays a 1,300 fold selectivity for CKII over CKI and has been shown to minimally affect a large number of other protein kinase activities [42]. Huh-7.5 cells were electroporated with transcripts of the infectious J6/JFH-1 RNA and DMAT was added to cell culture media four hours after RNA delivery. Following three days of further incubation, supernatants of these cultures were collected and used to infect naïve Huh-7.5 cells. Figure 4A shows IHC staining of the NS5A protein in these infected cells for two doses (0.5 µM and 2 µM) of DMAT and a DMSO only vehicle control. Robust infections of naïve cells is evident with the transfer of supernatants from the DMSO only treated cells, and a dose dependent reduction in infectious virion content is seen in infections using supernatants from DMAT treated cells. A more quantitative analysis of the effects of DMAT treatment on infectious virus production is presented in Figure 4B. For this experiment, cells were electroporated with J6/JFH-1 RNA transcripts, plated and incubated for four hours, and doses of DMAT were added to the cells. Following three days of incubation, cell supernatants were harvested for tittering on naïve cells. As observed in Figure 1A, a dose dependent inhibition of infectious virus release was observed with DMAT treatment, with no effect observed for DMSO vehicle only treatment. Infectious virus titers were plotted as the percent of infectivity release relative to the DMSO only treated cells versus the dose of DMAT. The EC_50_ (effective concentration 50%) of DMAT inhibition of infectivity release was calculated at 180 nM, in good agreement with the published IC50 (inhibitory concentration 50%) of DMAT of 140 nM as assayed on purified rat liver CKII [42]. Although these data suggested CKII activity was important for the production of infectious virions, it did not provide information about the phosphorylation of serine 457 of NS5A in this process. We therefore assayed the aspartic acid mutation at position 457 of NS5A in these experiments, based on the concept that aspartic acid should mimic phosphorylation of position 457 and make NS5A insensitive to CKII, and therefore, DMAT inhibition. The aspartic acid mutation at position 457 (Figure 4B, DEED) was insensitive to DMAT inhibition of infectivity release, with an EC_50_ value (5.5 µM, Figure 4B red line) close to the LD_50_ (lethal dose 50%) of DMAT on Huh-7.5 cells (6.5 µM, Figure 4B dashed line), suggesting that the inhibition of infectivity release for the aspartic acid mutation was largely due to cytotoxic effects of drug treatment. The roughly 30-fold difference in EC_50_ values of DMAT on wild type NS5A versus the aspartic acid mutation implicates serine 457 phosphorylation as an important event in infectious virus production and suggests that other possible CKII sites in NS5A have little role in this process. The replication of wild type or DEED HCV RNA, as determined by monitoring levels of genomic RNA in cells by real-time PCR, was not affected by treatment with DMAT up to the level where DMAT toxicity was observed (Figure 4C). Treatment of cells with an unrelated nucleotide analog kinase inhibitor, 2-aminopurine, had no effect on infectious virus release when used either to treat cells prior to infection or when maintained during the entire course of the experiment (figure 4D). As the NS2 protein is known to be a key determinant in the production of infectious virus, and also documented to contain a CKII site that can regulate the stability of NS2 [43], we treated cells infected with the JC1 HCV virus [44] with the DMAT inhibitor and monitored the stability of NS2 over the course of our previous measurements of infectivity (Figure 4E). The use of the JC1 virus, which alters the location of the junction between J6 and JFH-1 genomes in the region of NS2 was necessary, as we only have antibodies that recognize the JC1 form of NS2, and not that present in J6/JFH-1. In any case, the putative CKII site in NS2 is not different in these different viral constructs. We observed no difference in NS2 stability from 0.125 to 2 µM DMAT, a slight reduction in NS2 levels at 4 µM, and a nearly complete loss of NS2 at 6 µM. These data, combined with our observed EC_50_ for DMAT on infectious virus production for wild type NS5A bearing virus (180 nM), suggest that alterations in NS2 levels are not a significant factor in our experiments.

### Silencing casein kinase II expression disrupts virus production

**Figure 5:**
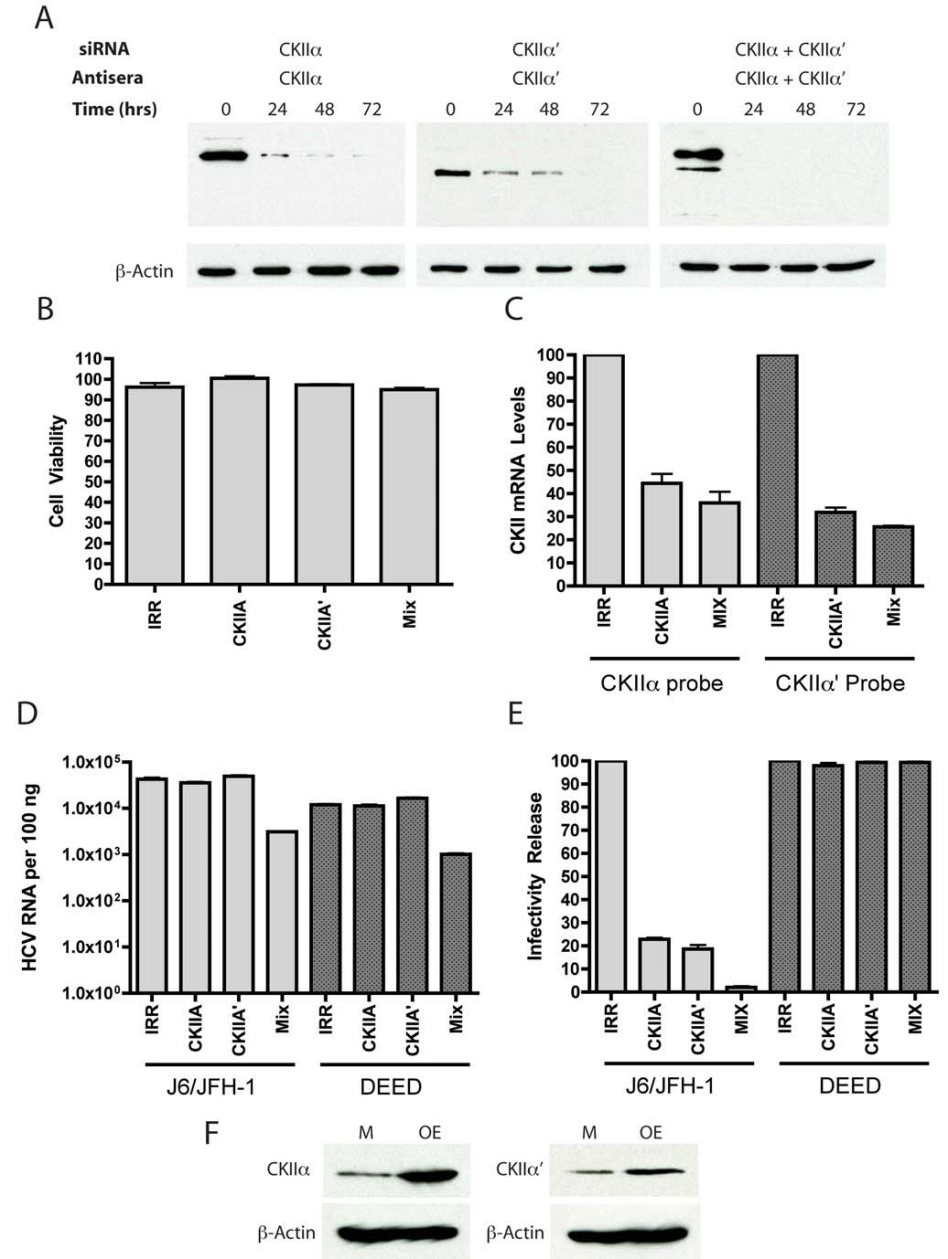
Levels of CKII α and α′ subunits affects infectious virus production. A). Western blots of cells electroporated with CKI α, CKIα′, or both CKIα and CKIα′ siRNAs are indicated and probed with antibodies recognizing CKI α, CKIα′, or both CKIα and CKIα′. A time course of 72 hours post siRNA delivery is shown. β-actin loading control panels are shown in the lower row of images. B). Cell viability, expressed as percent viability of cells electroporated with no siRNA, for cells electroporated with irrelevant siRNA (IRR), CKI α (CKIA), CKIα′ (CKIA′), or both CKIα and CKIα′ (MIX) as determined by ATP activity assay. C). CKI α and CKIα′ messenger RNA levels, relative to GAPDH in arbitrary units. Dark lines and text cues indicate which real time PCR probe set was used for each set of bars. Designations under bars indicate the siRNA delivered to the cells. Designations of siRNAs are as described in B. D). HCV RNA replication 72 hours post siRNA treatment for cells treated with various siRNAs (labels below columns) and infected with HCV viruses indicated below the dark bars (J6/JFH-1 or J6/JFH-1 with the DEED mutation). Column colors further highlight the different viruses used for these experiments, with light gray for J6/JFH-1 infected samples, and dark gray for J6/JFH-1 DEED infected samples. E). Infectivity release 72 hours post treatment with the indicated siRNAs (column labels) for cells infected with viruses indicated by dark bars (J6/JFH-1 or J6/JFH-1 with the DEED mutation). Column bar colors are as designated in D. F). CKII α and CKIIα′ catalytic subunit over-expression (OE) relative to cells transfected with an empty vector (M). Western blots using CKII α and CKIIα′ antisera as indicated. β-actin Western blot of samples shown in over-expression samples as a loading control.

Although the chemical inhibition of infectious virion production by the specific CKII inhibitor DMAT suggested CKII phosphorylates NS5A, the possibility existed that other kinases were inhibited by DMAT and were responsible for infectious virus production. To address this point we chose to specifically silence CKII gene expression. CKII exists as a tetramer composed of 2 β regulatory subunits and two α kinase subunits [45]. Two isoforms of CKII α subunits exist, designated α and α′. These α/α′ isoforms can assemble with 2 β subunits in a homotypic (all α or all α′ subunits in the CKII holoenzyme) or heterotypic manner (α and α′ in the CKII holoenzyme). As it was unclear which subunit might be involved in NS5A phosphorylation, we chose to silence both α and α′ expression individually as well as α′ and α simultaneously. siRNAs (CKII α, α′, a mix of α and α′, or an irrelevant siRNA) were delivered to cells via electroporation and the cells were plated and incubated for 24, 48 and 72 hours prior to harvesting protein lysates and total cellular RNA to determine the efficacy of silencing. Figure 5A shows Western blotting of cells treated with the various siRNAs using CKII α and α′ specific anti-sera. In all samples, a decrease in the amount of the targeting CKII alpha subunit(s) was evident within 24 hours, with little or no CKII α or α′detectable by 72 hours. To further confirm our silencing of the CKII kinase subunits we performed real-time reverse transcription PCR using primers and probes targeting CKII α and α′ mRNA (Figure 5C). We were able to reduce CKII α mRNA levels by about 60% relative to the level of an untreated control, and CKII α′ by approximately 65%. Cells treated with both CKII α and α′ RNAs (Figure 5C, Mix) showed similar silencing to cells treated with a single siRNA, although silencing was somewhat more efficient in cells receiving the mixture of both CKII siRNAs. Measurement of cell viability at 72 hours indicated that our siRNA treatments had little negative affect on the cells, with all cells at least 95% or better than the viability of untreated cells (Figure 5B). We therefore chose to conduct all future experiments 72 hours post siRNA delivery. Following siRNA electroporation and a 72 hour incubation, cells were infected with J6/JFH-1 or DEED virus at a multiplicity of infection of five. After five hours, the virus inoculum was removed and the cells washed extensively. After 48 hours of infection, supernatants were harvested and titered for the presence of infectious virus. J6/JFH-1 virus production was inhibited by silencing of either CKII α or α′, with reductions in virus yield of about 80% relative to an irrelevant siRNA control (Figure 5D). Silencing of CKII α and α′ together impaired wild type virus production by more that 98%. It should be noted that although silencing of CKII α or α′ individually did not significantly alter HCV RNA replication in these experiments, but silencing both CKII α and α′ together led to a one log reduction in HCV RNA levels (Figure 5D). This lower RNA replication may explain the observed increase in infectious virus inhibition in samples where both CKII α and α′ were silenced. The DEED mutation bearing virus infected cells were not inhibited in virus release when CKII α or α′ were silenced individually or in combination, in contrast to what was observed in J6/JFH-1 wild-type infections (Figure 5E). The DEED infections also had about a one log drop in HCV RNA in cells in which CKII α and α′ were both silenced (Figure 5D), but this did not alter virus production for these cells (Figure 5E). This, perhaps, not surprising as virus constructs such as JC1 are capable of increasing infectivity release without altering the levels of RNA replication, suggesting the relationship between replicating RNA and virion packaged RNA is not stoichiometric [44]. The siRNA data overall is similar to our use of the CKII inhibitor, DMAT, and collectively these experiments highlight the importance of serine 457 phosphorylation by CKII in the production of infectious virus.

### Over expression of CKII alpha increases virus production

Although chemical and genetic inhibition of CKII activity has suggested the importance of this kinase in the production of infectious virus for HCV, it remains a possibility that these treatments altered some other aspect of the host cell physiology. We therefore chose to over express the α and α′ subunits of CKII from a CMV promoter based plasmid and assay the effects of this on infectious virus release. The expression of CKII α has been shown to increase the production of the regulatory β subunit in cells, making delivery of exogenous CKII β not necessary [46]. We transiently transfected cells with pCMVSport6 CKII α, pCMVSport6 CKII α′, or a pCMVSport6 empty vector control, and following a 24 hour incubation to allow for expression of CKII α subunits, cells were infected with J6/JFH-1 virus. A Western blot image showing the level of CKII alpha subunit relative to a β-actin loading control are shown in Figure 5F. After 48 hours, cell culture supernatants were titered for the presence of infectious virus on naïve Huh-7.5 cells. Table 2 presents the results of this experiment, with values listed as fold increase in infectious virus release relative to a non-plasmid transfected cell control. The presence of the pCMVSport6 control vector had no affect on the production of infectious virus, but the expression of CKII α from this vector led to a 3.44 fold increase in infectious J6/JFH-1 virus yield. Similarly, CKII α′ led to a 2.95 fold increase in virus titers. We also conducted a parallel experiment in which similarly transfected cells were infected with a J6/JFH-1 DEED mutant virus. The DEED mutant virus showed no increase in virus production with the over expression of CKII α (0.83 fold change) or CKII α′ (0.94 fold change) (Table 2). These data highlight the importance of CKII to infectious virus production, and in the case of the DEED mutant virus, the importance of a single residue of the NS5A protein to this process.

**Table 2:** CKII over expression increases infectious virus release.

Sample^a^	Fold Increase in Virus Titer^b^
J6/JFH-1+vector	0
J6/JFH-1+CKIIα	3.44 (+/−0.21)
J6/JFH-1+CKIIα′	2.95 (+/−0.28)
J6/JFH-1 DEED+vector	0
J6/JFH-1 DEED+CKIIα	0.83 (+/−0.23)
J6/JFH-1 DEED+CKIIα′	0.94 (+/−0.32)

a Sample column organized as “infecting virus+input expression construct”.

b Values reported as average of three independent experiments in the format “average value (+/−standard deviation)” increase relative to non-transfected control.

### Serine 457 is phosphorylated by CKII in vitro

**Figure 6:**
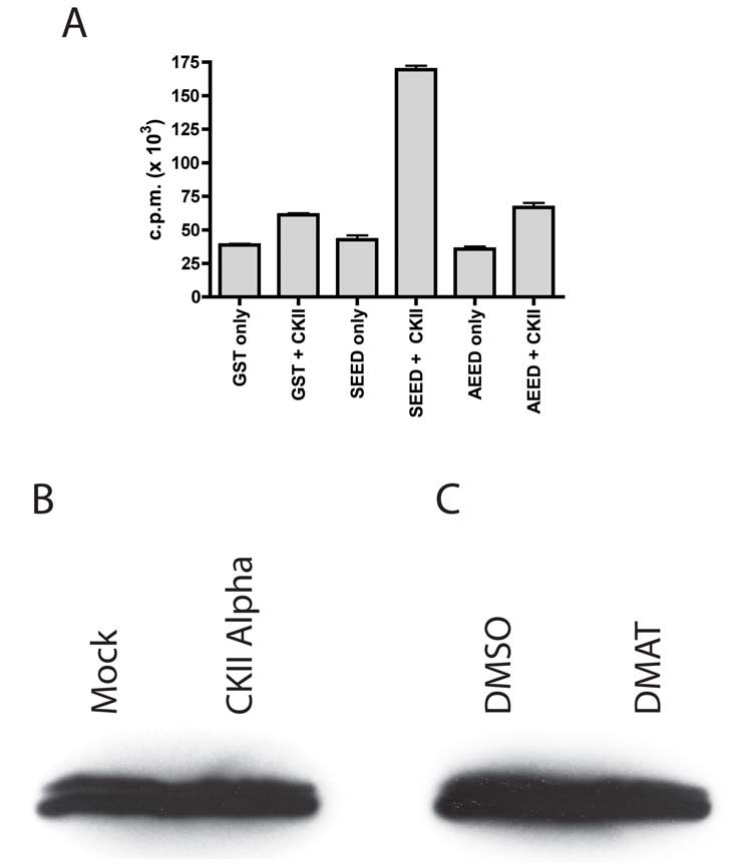
Serine 457 is phosphorylated by CKII in vitro and in vivo. A). Results of in vitro phosphorylation analysis of GST fusion proteins of the region of NS5A surrounding serine 457 presented as counts per minute (c.p.m.) of γ-^32^P incorporation. A non fusion protein form of GST assayed in the absence (GST only) and presence of recombinant human CKII (GST+CKII) serve as negative controls. GST fused to the NS5A derived sequence SGSGWSTCSEEDTTVV, which corresponds to a wild type sequence surrounding serine 457, was assayed in the absence (SEED only) and presence of CKII (SEED+CKII). GST fused to the NS5A derived sequence SGSGWSTCAEEDTTVV, which replaces serine 457 with an alanine residue but otherwise retains a wild type NS5A sequence, was also analyzed for phosphate incorporation in the absence (AEED only) and presence (AEED+CKII) of purified CKII. B). Results of CKII over-expression on NS5A phosphorylation during a virus infection. Cells were transfected with an empty expression vector construct (Mock) or a construct expressing human CKII α, lysed, and lysates probed with the 9E10 anti-NS5A monoclonal antibody. C). Analysis of DMAT treatment on NS5A phosphorylation. DMAT or vehicle (DMSO) was added to cells at a concentration of 250 nM for a period of two days. Cell lysates were than analyzed as described for B.

Although our genetic and chemical manipulations of NS5A suggest serine 457 is phosphorylated by CKII, we have not shown direct modification of this residue by this kinase. Indeed, only two sites of NS5A phosphorylation have been mapped despite intense efforts in this regard over the past 15 years [47],[48]. This is, at least in part, due to the large number of potential acceptor sites in NS5A and the unusual repetitive amino acid usage of potential acceptor sites in several regions of NS5A. Our efforts in attempting to identify serine 457 as a phosphoacceptor from NS5A isolated from infected cells by immunoprecipitation and mass spectrometry have not been successful due to poor coverage of the domain III region of NS5A in our spectra (data not shown). We therefore decided to analyze the deletion B region as a potential CKII substrate using in vitro kinase reactions on GST fusion protein substrates. We generated two GST fusion proteins for use as in vitro substrates for CKII phosphorylation. The first contained amino acids 449 to 465 (GSGSWSTCSEEDDTTVV, in single letter amino acid designation) from the JFH-1 NS5A protein fused to the C-terminus of GST. The second fusion protein was identical with the exception of the substitution of serine 457 with an alanine residue (GSGSWSTCAEEDDTTVV). Following incubation with recombinant human CKII in the presence of γ-^33^P ATP, we captured the proteins on glutathione agarose beads and determined radiolabel incorporation by liquid scintillation counting. This data is presented in Figure 6. Proteins were first analyzed in the absence of added CKII to assess the level of phosphate incorporation that might be present from co-purifying kinase activities. All of the proteins showed similar levels of incorporation in these reactions, indicating that the purified proteins were essentially equivalent in regard to potential kinase contamination. The fusion protein containing serine 457 (designated SEED in Figure 6) incorporated about 3 times more radiolabel than GST with no fusion in the presence of recombinant CKII, indicating residues in the fusion sequence were modified by CKII. The fusion protein in which serine 457 was changed to an alanine residue, but was otherwise identical to the SEED fusion protein, did not show this increased label incorporation relative to the GST control, suggesting that the modification of the SEED fusion protein involved serine 457. These data confirm that serine 457 is a valid CKII target, at least in these in vitro reactions. We have attempted to detect the effects of CKII α over-expression and the DMAT inhibitor on NS5A phosphorylation in the context of an HCV cell culture infection. Unfortunately, we observe only a very small effect on NS5A hyperphosphorylation with CKII α over-expression (Figure 6B), and only a very moderate decrease in hyperphosphorylation (Figure 6C) with these treatments. These data are far less dramatic that our previous genetic manipulations, suggesting more advanced phosphopeptide mapping will be required to provide a definitive answer on whether NS5A phosphorylation is altered by these treatments.

### Deletion B does not disrupt localization of NS5A to lipid droplets

**Figure 7:**
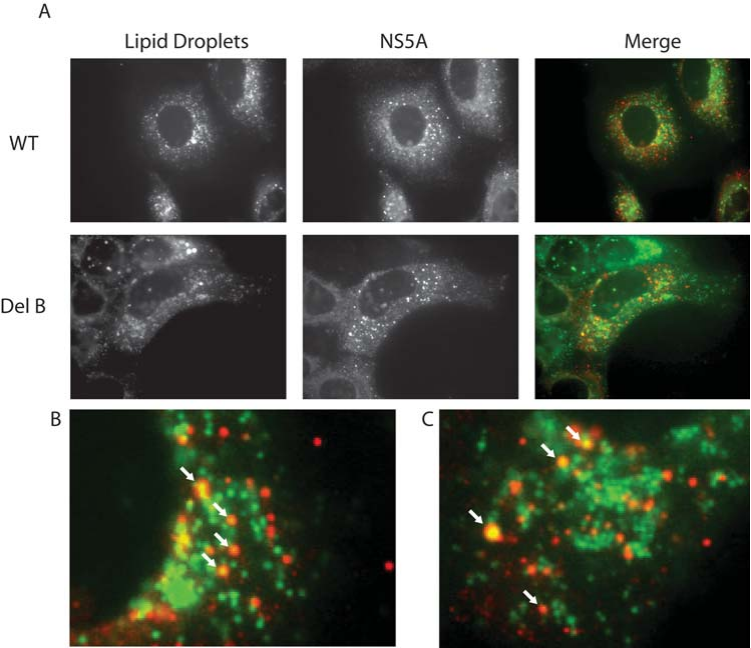
Deletion B does not alter the localization of NS5A to lipid droplets. A). Immunofluorescence microscopy of lipid droplets and NS5A from wild type J6/JFH-1 (top row) and a J6/JFH-1 virus bearing the deletion B mutation (bottom row). A merged color image of the lipid droplets and NS5A stained images is also shown (merge). B. A close up of a region of the wild type J6/JFH-1 merged image shown in A. White arrows indicate lipid droplets coated with NS5A. C. A close up of a region of the deletion B bearing J6/JFH-1 merged image shown in A. Arrows indicate lipid droplets coated with NS5A.

Recent research has implicated the lipid droplet as an important organelle in the production of HCV virions [7]. The localization of HCV core to lipid droplets, and the subsequence recruitment of viral RNA and non-structural proteins are prerequisites of virion production. A mutation in the domain I region of NS5A that disrupts lipid droplet association was shown to prevent the production of virions in this work. The localization of our deletion B bearing NS5A to lipid droplets was therefore of significant interest. Using a lipid droplet specific fluorescent dye and NS5A indirect immunofluorescence microscopy we compared the localization of wild type and deletion B NS5A proteins in the context of cells electroporated with replication competent full-length viral genomes. Figure 7 shows the results of this analysis, with panels showing lipid droplet and NS5A staining, as well as merged images to examine co-localization. Panels B and C show close up views of this analysis for wild type and deletion B bearing genomes, respectively. In the case of both mutant and wild type NS5A proteins, we see localization of NS5A to lipid droplets. We were unable to observe any difference in the localization of NS5A in any of our efforts in this regard, suggesting that deletion B is functionally quite different than the NS5A mutant that blocks infectivity release by disrupting localization to lipid droplets [7].

## Discussion

The idea of NS5A as a regulator of events in the HCV lifecycle via phosphorylation by cellular kinases is not a new concept. Indeed, this idea has been prevalent in the HCV community since the discovery that NS5A exists in multiple phosphorylation states [21]. Considerable progress has been made in deciphering the role of CKIα phosphorylation of NS5A in RNA replication [22]. The functional significance of CKII phosphorylation of NS5A, however, has long been an enigma. Our data provides the first evidence for a function of these events, not in RNA replication, but in the process of virion assembly.

We have shown that CKII likely phosphorylates serine 457 of genotype 2a NS5A, and this modification is required for infectious virus production. Generating an NS5A that is incapable of serving as a phosphoacceptor (alanine mutation at position 457) does not alter RNA replication, but blocks the production of infectious virus. Generating an aspartic acid mutation at position 457 lowers RNA replication slightly but does not affect infectivity release. This lack of direct coupling of replication levels and infectivity release is in agreement with that observed for the JC1 chimeric virus [44], in which infectivity is increased ten fold with no change in RNA replication levels. Our first interpretation of this data was that phosphorylation of serine 457, mimicked by the aspartic acid mutation, destabilized the replicase leading to release of NS5A and viral RNA from the replicase for assembly events. This idea is roughly equivalent to the models of phosphorylation as a regulator of the replicase first proposed by Evans, et al. [26],[44]The case is clearly not this simple for our mutants, as we see NS5A participating in later events in assembly, such as association with lipid droplets even when serine 457 (and surrounding residues) are deleted. In this regard, our data is quite different than the recently described NS5A mutants in domain I that block infectious virus production by hindering the association of NS5A with lipid droplets [7]. These mutations are severely impaired in RNA replication, yet release what appear to be non-infectious virus particles containing both HCV core and viral RNA. In contrast, our deletions and single amino acid serine 457 to alanine mutant in domain III does not alter HCV RNA replication, but blocks the production of infectious virus, and does not release large amounts of viral RNA and HCV core. We see no defect in the association of NS5A with lipid droplets with our deletion B mutant, further highlighting the difference between domain I and domain III mutants in NS5A assembly activities. Collectively, these data suggest our mutants affect a different step in the HCV assembly pathway than the domain I mutations [7]. Our lack of understanding of the mechanisms of HCV assembly make it difficult to speculate exactly what step in assembly our mutations are affecting. We are clearly manipulating an early step in the assembly process, as we do not see virus particles inside the cell secretory pathway, suggesting, in conjunction with the lack of RNA and core release, that particles are simply not generated. The data suggests that we are affecting a later stage in assembly than the domain I mutants, as we see clear evidence of lipid droplet association of NS5A in the context of our mutant. What this later step of assembly might be is unknown. Perhaps it involves NS5A making productive interactions with the structural proteins at the lipid droplet, NS5A moving and releasing viral RNA, NS5A functioning to package viral RNA, or NS5A directly being packaged into virions as a minor component of the infectious virion. Unfortunately, our understanding of the properties of NS5A is still in its infancy, making directly addressing these issues difficult at the present time.

Although our genetic manipulations of NS5A suggest serine 457 is phosphorylated by CKII, and we can show phosphorylation of fusion protein substrates corresponding to this region of NS5A, we have not shown direct modification of this residue by this kinase in the context of an authentic virus infection. Indeed, only two sites of NS5A phosphorylation have been mapped to date, and not in the context of authentic RNA replication, despite intense efforts in this regard over the past 15 years [47],[48]. This is, at least in part, due to the large number of potential acceptor sites in NS5A and the unusual repetitive amino acid usage of potential acceptor sites in several regions of NS5A. Our efforts in attempting to identify serine 457 as a phosphoacceptor from NS5A isolated from infected cells by immunoprecipitation and mass spectrometry have not been successful due to poor coverage of the domain III region of NS5A in our spectra (data not shown). Based on the lack of evidence in the literature of documented NS5A phosphorylation sites, and our lack of understanding of what the differences in phosphorylation that generate the different phosphoforms of NS5A, it will likely be some time before the complete complement of NS5A phosphorylation sites are known. Even understanding whether this site is a component of the hypo or hyperphosphorylated form of NS5A is not a simple task. Serine 457 lies in a region of NS5A domain III that is believed to primarily contain sites of hypophosphorylation based on alanine scanning mutagenesis of potential acceptor sites in domain III, although this particular residue position has never been evaluated by mutagenesis in the context of the 2a NS5A sequence [11]. Our Western blot data suggests serine 457 may either be a hyperphosphorylation site, or a residue that when phosphorylated, promotes hyperphosphorylation of NS5A. The later seems more likely, as aspartic acid mutations at serine 457 have normal levels of hyperphosphorylation, despite being incompetent as an acceptor site. The work of Miyanari, et al. shows that the lipid droplet associated form of NS5A, a requirement for virus assembly, is enriched for hyperphosphorylated NS5A relative to what is seen in viruses lacking the core protein, suggesting hyperphosphorylation might be important in assembly. This is, at least in broad terms, supported by the observation that hyperphosphorylated NS5A destabilizes the replicase. Nonetheless, in vitro phosphorylation reactions of NS5A with recombinant CKII only generate the hypophosphorylated form of NS5A, even in conditions of excess CKII [25]. At least for CKI phosphorylation of NS5A in vitro, several acceptor sites require activation by pre-phosphorylation of upstream sites before they are modified, suggesting that in vitro reactions probably do not fully represent what occurs in vivo [25]. Although we have shown serine 457 is an important residue in infectious virus production, it may be that this residue merely ‘activates’ other phosphoacceptor sites, as has been observed for CKIα phopshorylation. This concept fits well with our observations of altered levels of hyperphosphorylation with our mutants.

Although the cell culture infectious genotype 2a strain clearly possesses an insertion relative to other HCV genotypes (Figure 1A), it is unlikely that this insertion is directly related to the ability to generate infectious virus. Clearly, isolates of genotype 1a are infectious in cell culture, but lack this insertion [34]. Indeed, we can delete this region without disrupting infectious virus production. The conservation of a CKII site around residue 457 seems to be a more likely candidate for an important conserved feature, with other defects preventing the production of infectious virus in cell culture for other genotypes. In this regard, it is worth noting that the web based NetPhosK 1.0 server [41] can predict CKII sites in all 35 HCV genotype reference sequences in the Los Alamos HCV database [49] that involve direct serine 457 modification. A recent paper looking at the sequence of patient isolated HCV RNA for genotypes 1a, 1b and 3 identified a number of potential CKII sites in NS5A, including sites very close to the location of serine 457 in genotype 2a, however the majority of the sites predicted have residues corresponding to serine 457 as sites of modification by another enzyme that generate a CKII recognition site just upstream of this position [50]. This analysis was based on the site motif described by Pinna [51], in which most CK2 sites typically have a serine or threonine, followed by an acidic residue at the n+3 position. This work did not take into account, as mentioned by Pinna, that whenever the negatively charged determinant is absent at position *n*+3, it is invariably present at position *n*+1 and vice-versa, leading to some variability in CK2 sites [51]. The majority of sites we predicted as conserved across all HCV reference strain had met these rules of the more conventional n+3, but many were of the less common n+1 variety. We also were able to predict the sites using an upstream CK2 site with serine 457 serving as an activator when phosphorylated, as described by Dal Pero and colleagues [50]. Unfortunately, it will likely be some time before the intricacies of NS5A phosphorylation, RNA replication, and now virion assembly, will be fully understood, thereby addressing the question of what type of CK2 sites are utilized in this region of NS5A. The lack of solid information on the regulation of CKII further complicates our understanding of when CKII might be active on NS5A and at what location in the infected cell (see [52] for review).

The requirement for phosphorylation of a viral non-structural viral replication protein by a host cell kinase for the production of infection virus particles, although novel for HCV, has been described previously for other viruses. Bluetongue virus (BTV) is perhaps the closest example of this phenomenon to what we observe in the case of HCV, despite the great evolutionary distance between these viruses. The BTV NS2 protein is phosphorylated by CKII, and this phosphorylation is required for the assembly of the site of replication virion assembly, termed viral inclusion bodies [53]. As is the case for HCV, the precise mechanisms of this phenomenon are not fully understood in BTV biology. Viruses more closely related to HCV, such as the flaviviruses Dengue (DENV) and West Nile virus (WNV), require a functional c-Src (or c-Src like) kinase activity for the assembly and maturation of infectious virus particles at the site of RNA replication [54],[55]. The mechanism of these events is also unknown, but the relatively close relationship of DENV and WNV to HCV suggest c-Src and c-Src like activities and CKII may perform similar regulatory functions in switching between replication and assembly. It seems that the regulation of switching between replication and assembly by phosphorylation, or controlling later stages of assembly and mturation may be a common, but understudied mechanism in virology.

In summary, we have identified serine 457 of the HCV NS5A protein as a key determinant for the production of infectious virions and presented data showing this site is likely a target for CKII phosphorylation. Phosphorylation of this residue appears to be an important regulatory step in infectious virus production. Via genetic and chemical manipulations we have shown that HCV RNA replication can be uncoupled from infectious virus production without altering HCV RNA replication efficiency. It remains to be demonstrated whether these observations are valid in the context of an infected liver, but at least in cell culture these events appear to be a critical step in the virus lifecycle. Nonetheless, these new genetic and chemical tools, when combined with recently identified mutants in NS5A that alter localization to the site of virion assembly, will be of significant value in further dissection of the mechanisms of HCV RNA replication and virion biogenesis.

## Materials and Methods

### Clones, Cells, and Antibodies

The construction of the J6/JFH-1, J6/JFH-1 pol-, H77/JFH-1, and pSGR-JFH-1 replicon have been described previously [31]. We generated these clones using a J6/JFH-1 chimeric virus backbone generated in house by oligo based gene synthesis. Deletions and mutations in NS5A were generated using the QuikChange mutagenesis system following the manufacturer's instructions. All constructs, subclones, and mutants were confirmed by DNA sequence analysis. The pCMVSport6 vectors encoding the human CKII α and α′ cDNA sequences were obtained from Dr. Michael Conkright. Huh-7.5 (human hepatoma) cells were obtained from an original seed stock of this clone and cultured as described previously [35]. The anti-human CKII α (A300-197A) and CKII α′ (A300-199A) purified rabbit polyclonal antibodies were obtained Bethyl Labs. The anti human β-actin mouse monoclonal loading control antibody (A-5441) was obtained from Sigma Aldrich. The anti-NS5A 9E10 antibody has been described previously [31]. Secondary antibody horseradish peroxidase conjugates were obtained from Pierce Biotechnology.

### Electroporation of Viral RNAs

Viral RNAs were generated and purified as described previously [31]. For electroporation, subconfluent monolayer cultures of Huh-7.5 cells were trypsinized, washed twice in ice cold RNase free phosphate buffered saline (Cambrex, Bio-Whitaker), and resuspended at a concentration of 1.25×10^7^ cells/ml in ice cold RNase free PBS. A 400 µl aliquot of cells was then mixed with 1 µg of HCV viral RNA and electroporated using a BTX ECM830 square wavelength electroporator in a 2 mm gap cuvette. Electroporation conditions were 5 pulses of 920 volts for 99 microseconds with 1.1 second intervals. Following a ten minute recovery time, cells were removed form the cuvette and diluted into 13.6 milliliters of DMEM +10% FBS.

### Virus Replication and Infectivity by Immunohistochemistry and Virus Titering

Monitoring virus replication and infectivity by IHC was performed as described previously [31], with minor modifications. Briefly, Huh-7.5 cells electroporated with HCV RNA were plated and incubated for 48 hours. Cell supernatants were then harvested and filtered through a 0.2 micron filter and stored for infection of naïve cells. The cell monolayers were then washed twice in PBS and fixed in methanol. For infection studies, naïve cells were inoculated with the filtered supernatant. Following a six hour incubation, the inoculum was replaced with fresh DMEM +10% FBS and cells were incubated for a total of 48 hours. Cells were then washed and fixed in methanol. Fixed cells were then probed for NS5A expression using the 9E10 monoclonal antibody and procedure described previously [31]. The limiting dilution virus titering assay was performed as described previously [31].

### HCV RNA quantitation

For measuring HCV RNA levels in cell lysates following various experimental treatments, cells were washed twice in PBS and RNAs were then purified using the Qiagen RNeasy kit using the manufacturer's instructions. For measuring HCV RNA levels in cell culture supernatants, electroporated cells were plated for 24 hours, washed 3 times in PBS, trypsinized/split 1∶2, and plated for an additional 48 hours. Supernatants were then harvested and RNA extracted using the Qiagen Qia Amp UltraSens Virus kit using the manufacturer's instructions. Extracted, purified RNAs were quantified by UV absorbance and 100 nanograms of total RNA was used for real time reverse transriptase PCR. All real time PCR experiments were performed using a Roche lightcycler 480 using HCV specific primers and probe as described previously [39]. Values are normalized using the human GAPDH endogenous control primer set (Applied Biosystems, # 4326317E).

### Intracellular virus release

Huh-7.5 cells were electroporated with the appropriate HCV RNAs as described herein. 5×10^5^ electroporated cells were plated in a 35 mm dish in DMEM +10% FBS and incubated at 37°C for 2 days. Culture media was then removed, the cells were washed twice in PBS and trypsinzed. The cells were then collected by centrifugation at 5,000× g for 5 minutes and resuspended in 2 ml of DMEM +10% FBS and lysed by four rounds of freezing and thawing. Cell lysates were then centrifuged at 5,000× g for 5 minutes, filtered through a 0.2 micron filter and buffered with the addition of HEPES pH 7.4 to a final concentration of 20 mM.

### DMAT treatment

2-dimethylamino-4,5,6,7-tetrabromo-1H-benzimidazole (DMAT) was purchased from EMD Biosciences and dissolved in sterile dimethyl suffixed (DMSO). Following the electroporation of J6/JFH-1 or J6/JFH1 DEED mutant RNA, cells were plated and incubated at 37°C for four hours. Cell culture media was then replaced with media supplemented with DMAT in DMSO or DMSO only. Cells were then incubated for 48 hours. Cell supernatants were then collected, filtered through a 0.2 micron filter, and titered for the presence of infectious virions. Cell lysates were collected for determination of HCV RNA levels. Cell viability was determined using the Celltiter-Glo assay on DMAT treated or control DMSO only treated cells. For experiments using the control compound, 2-aminopurine (2-AP), cells were treated with 2-AP for one hour prior to infection, washed extensively, and infected with a high titer stock of J6/JFH-1 virus. In some experiments, 2-AP was left in the culture medium at 1 mM (maximum concentration without toxicity) for the course of the experiment. Output virus was harvested and titered by limiting dilution. To determine the effects of DMAT inhibition of NS2 stability, cells electroporated with JC1 viral RNA were treated with DMAT for 48 hours, lysed and probed for NS2 using the 6H6 monoclonal antibody. β-actin was used as a loading control for these experiments.

### CKII siRNA experiments

Pre-annealed, validated Silencer siRNA mixtures targeting human CKII α (siRNA # 1337) and α′ (siRNA # 183) and an irrelevant siRNA (siRNA # 4611) were purchased from Ambion. Electroporation of siRNAs was performed as described previously [56], with minor modifications. One nanomole of RNA duplexes was electroporated with 2.5×10^6^ Huh-7.5 cells in 0.4 ml of RNase free PBS. Electroporation conditions were the same as used for HCV genomic RNA delivery. Cells were infected 72 hours after electroporation with a multiplicity of 5 for 6 hours, rinsed with media, then maintained for 2 days at 37°C. Cell supernatants and lysates were then harvested for assessment of HCV replication (infectious particles released and intracellular RNA level) and cell viability (Celltiter-Glo assay, Promega).

### CKII messenger RNA quantitation

Real-time reverse transcriptase PCR analysis of CKII gene expression was performed using Applied Biosystems pre-designed Taqman assays and total cellular RNA prepared as described for HCV specific real time PCR analysis. CKII α was assayed using assay number Hs00601957_m1. CKII α′ was assayed using assay Hs00176505_m1. Values were normalized for RNA level using the expression of human GAPDH (Applied Biosystems, # 4326317E).

### CKII Over-expression Studies

Huh-7.5 cells were transfected with pCMV-Sport6 vectors expressing CKII α, α′, or an empty vector control using Fugene 6 transfection reagent. Following a 24 hour incubation, cells were infected with J6/JFH-1 virus for 4 hours, washed, and incubated a further 48 hours, at which point supernatants were collected for virus titering. The levels of CKII α and α′ in these experiments were demonstrated by Western blotting of cell lysates using CKII specific anti-sera as described above. β-actin was used as a loading control in these experiments.

### HCV Core Protein ELISA

For core protein release assays, Huh-7.5 cells were electroporated with J6/JFH-1, J6/JFH-1 deletion B, or J6/JFH-1 pol- RNA. Supernatants were harvested at 24 hours post transfection and HCV core protein was measured using the Ortho Diagnostics Trak-C HCV core ELISA kit following the manufacturer's instructions.

### GST Fusion Protein Production and Phosphorylation

To generate GST fusion proteins of the deletion B region of NS5A, complementary 5′ phosphorylated oligos were generated coding for amino acid 449–465 of the JFH-1 NS5A protein with either a serine or an alanine at position 457. The oligos we designed such that when annealed they generated appropiate ends for direct cloning into a pGEX6p1 vector previously digested with BamHI and EcoRI. The resulting pGEX6p1 vectors encoded deletion B fused to the C-terminus of GST. After DNA sequence analysis to confirm the clones were correct, we transferred these vectors to E.coli strain BL21 and induced the production of the fusion protein with IPTG. Briefly, 1 liter LB broth cultures of BL21 cells with the appropriate fusion protein encoding vector were grow at 37°C until an OD_600_ of 0.6 was reached. Cells were then induced by the addition of 1 mM β-2-D-isopropyl-thio-galactoside and grown for an additional 4 hours at 37°C. Cells were then pelleted, resuspended in 20 ml of cold buffer A (25 mM Tris-HCl pH 8.0, 25 mM NaCl, and 20% glycerol) and lysed by three passes through an Avestin air emulsifier. Lysates were clarified by centrifugation at 25,000× g for 20 minutes at 4°C, and the soluble fractions were loaded onto a 5 ml HiTrap GST FF column equilibrated in buffer A. After extensive washing of the column to remove unbound material, the fusion proteins were eluted with buffer A supplemented with 10 mM reduced glutathione. The reduced glutathione was then removed by desalting the proteins into buffer A using a HiPrep 26/10 desalting column. The purity of proteins was assessed by SDS-PAGE and concentrations were determined by Bio-Rad protein assay. For in vitro phosphorylation experiments 25 µl reactions were prepared containing 2 µM of each fusion protein, 100 µM unlabeled rATP, 5 µCi of γ-^32^P-rATP, in 1× CKII kinase reaction buffer (New England Biolabs). Phosphorylation was initiated by the addition of 500 units of recombinant human CKII (New England Biolabs), and reactions were subsequently incubated at 30°C for 30 minutes. After incubation, 100 µl of PBS was added to the reaction, followed by 50 µl of a 50% slurry of glutathione 4B agarose (GE Healthcare) equilibrated in PBS. Proteins were allowed to bind to the agarose beads for 10 minutes at 4°C, the centrifuged at top speed in a microfuge, and the unincorporated label (supernatant) was removed. This pelleting and washing step was repeated 4 times. After washing, pellets were resuspended in 100 µl of PBS and transferred to a scintillation vial with 10 ml of aqueous scintillation cocktail and counted using a Beckman liquid scintillation counter.

### NS5A and Lipid Droplet Visualization

To visualize lipid droplets and NS5A, cells were electroporated with 1 µg of the appropriate HC RNA, plated in glass slide micro-chambers, incubated 48 hours, fixed by a 20 minute incubation in 4% paraformaldehyde and probed with 5 µg/ml BODIPY 493/503 (Invitrogen/Molecular Probes) for lipid droplet staining and a 1∶15,000 dilution of the 9E10 monoclonal anti-NS5A antibody and an Alexa 568 secondary antibody (Invitrogen/Molecular Probes). Slides were then mounted in Vectashield (Vector Labs) soft mounting medium and visualized.
